# Supplementary material for: The C-Terminus of the PSMA3 Proteasome Subunit Preferentially Traps Intrinsically Disordered Proteins for Degradation
Source: Cells. 2022 Oct 14;11(20):3231. doi: 10.3390/cells11203231 (PMC9600399; doi:10.3390/cells11203231)
Supplement: Supplementary file 1 [file cells-11-03231-s001.zip › cells-1883910-supplementary.pdf]

Supplementary Information for

The C-terminus of the PSMA3 proteasome subunit preferentially trapsin-trinsically disordered proteins for degradation

Assaf Biran, Nadav Myers, Shirel Steinberger, Julia Adler, Marianna Riutin, Karin Broennimann, Nina Reuven and Yosef Shaul*

Yosef Shaul yosef.shaul@weizmann.ac.il

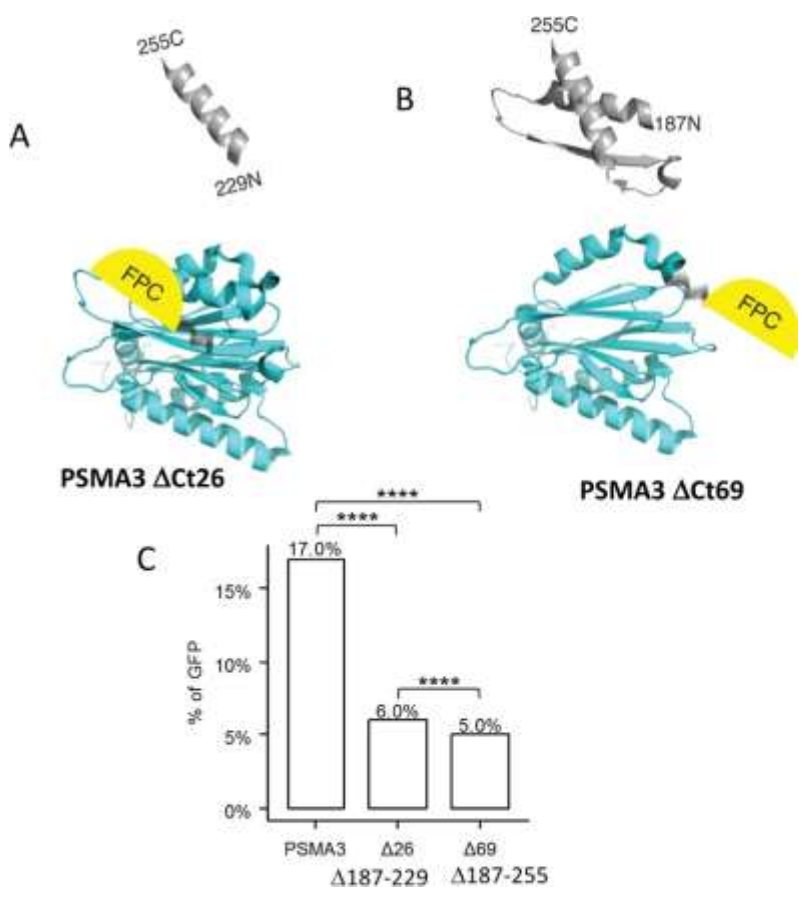

Figure S1. The PSMA3 C-terminus binds p21. (A) Shows the predicted structure of PSMA3 ◎26 C-terminus mutant and the structure of the deleted region. (B) As in A, but the PSMA3 ◎69 C-terminus is described. (C) HEK293 cells were transiently transfected as indicated with 6xmyc p21 FPN, chimeric PSMA3 WT, deletion mutants and H2B RFP constructs. The latter monitors for the transfected cells. The percent of the GFP positive cells is compared between the different constructs.

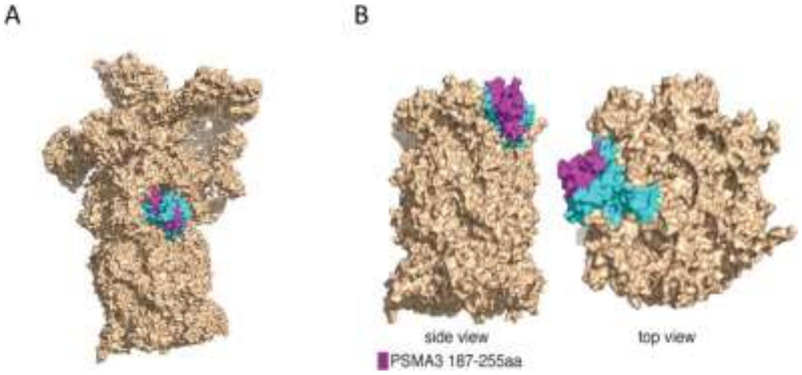

Figure S2. PSMA3 C-terminus is exposed in the context of the 20S and 26S proteasome complexes.

(A) Cryo-EM structure of the 26S proteasome marking PSMA3 Ct. (B) Structure of the 20S proteasome marking PSMA3 Ct. The indicated C-terminal portions are labeled in magenta, and remaining PSMA3 and PSMA5 subunits are labeled in cyan and green, respectively.

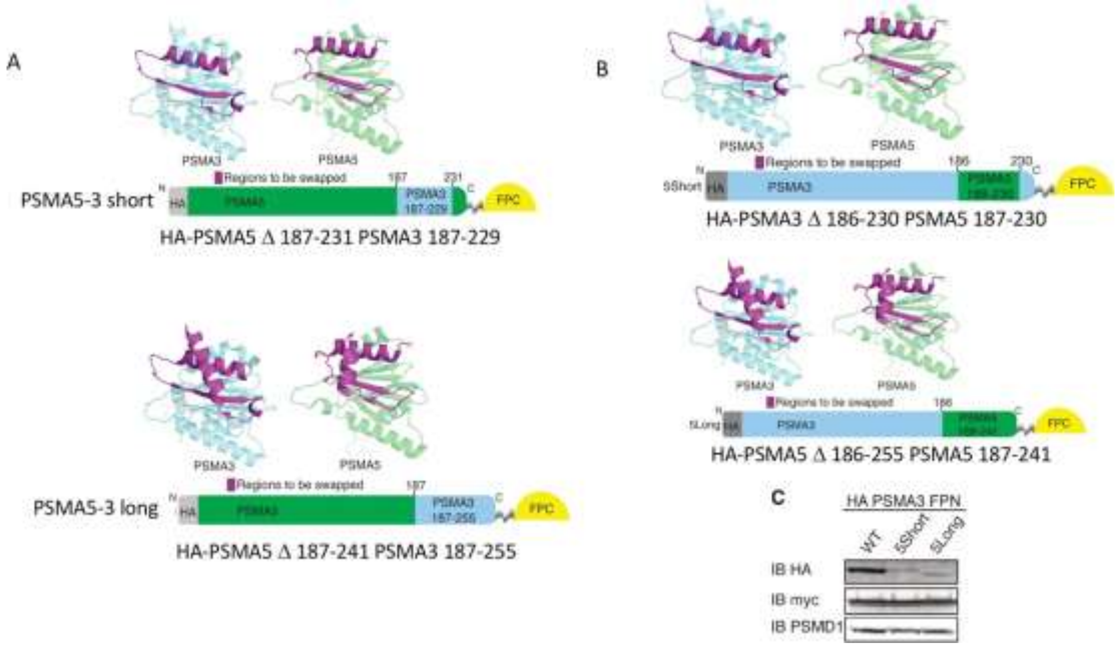

Figure S3. PSMA5 and PSMA3 chimeric constructs. (A) Illustration of chimeric constructs of PSMA5 and PSMA3 C-terminus region used in our experiments. (B) Illustration of the chimeric constructs of PSMA3 and PSMA5 C-terminus region is shown. (C) The expression level of the constructs shown in B.

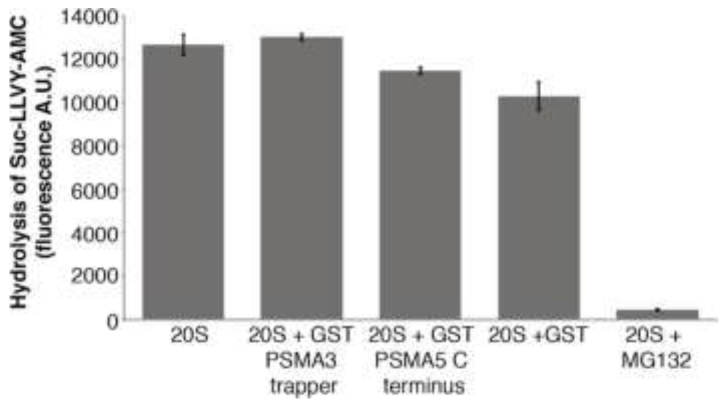

Figure S4. The PSMA3 trapper does not inhibit proteasome catalytic activity. Purified 20S proteasome was incubated for 30 min at 37◦C as indicated with purified GST, GST PSMA3 trapper, GST PSMA5 C-terminus and proteasome inhibitor MG132 in the presence of the chymotrypsin-like fluorogenic substrate Suc-LLVY-AMC. Standard deviation bars represent three independent experiments (N=3).
